# A nonsense mutation in *MME* gene associates with autosomal recessive late‐onset Charcot–Marie–Tooth disease

**DOI:** 10.1002/mgg3.1913

**Published:** 2022-02-25

**Authors:** Zeinab Jamiri, Rana Khosravi, Mohammad Mehdi Heidari, Ebrahim Kiani, Javad Gharechahi

**Affiliations:** ^1^ Department of ‌Biology, Faculty of Science Yazd University Yazd Iran; ^2^ Department of Biology, Faculty of Science University of Zabol Zabol Iran; ^3^ Human Genetics Research Center Baqiyatallah University of Medical Sciences Tehran Iran

**Keywords:** Charcot–Marie–Tooth disease, membrane metalloendopeptidase, polyneuropathy, Sanger sequencing, whole exome sequencing

## Abstract

**Background:**

The genetic cause for the majority of patients with late‐onset axonal form of neuropathies have remained unknown. In this study we aimed to identify the causal mutation in a family with multiple affected individuals manifesting a range of phenotypic features consistent with late‐onset sensorimotor axonal polyneuropathy.

**Methods:**

Whole exome sequencing (WES) followed by targeted variant screening and prioritization was performed to identify the candidate mutation. The co‐segregation of the mutation with the phenotype was confirmed by Sanger sequencing.

**Results:**

We identified a nonsense mutation (c.1564C>T; p.Q522*) in membrane metalloendopeptidase (*MME*) gene as the cause of the disease condition. The mutation has a combined annotation‐ dependent depletion (CADD) score 45 and predicted to be deleterious based on various algorithms. The mutation was inherited in an autosomal recessive mode and further confirmed to co‐segregate with the disease phenotype in the family and showed to has the required criteria including rarity and deleteriousness to be considered as pathogenic.

**Conclusion:**

The *MME* gene encodes for the membrane bound endopeptidase neprilysin (NEP) which is involved in processing of various peptide substrates. The identified mutation causes a complete loss of carboxy‐terminal region of the NEP protein which contains the zinc binding site and the catalytic domain and thus considered to be a loss‐of‐function mutation. The loss of NEP activity is likely associated with impaired myelination and axonal injury which is hallmark of CMT diseases.

## Graphical abstract

We applied whole exome sequencing to identify the causative mutation for a late‐onset axonal form of polyneuropathy. The candidate mutation was a nonsense mutation in *MME* gene which causes a complete loss of zinc binding and active site of the neprilysin protein. The mutation was co‐segregated with the disease and has the required criteria including rarity and deleteriousness to be considered as pathogenic.

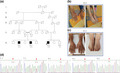

## 1 INTRODUCTION

Charcot–Marie–Tooth disease (CMT) constitutes a large clinically and genetically heterogenous groups of inherited neuropathies characterized by their progressive loss of peripheral nerves resulting in muscle weakness in the distal parts of limbs including hands and feet. Degeneration of peripheral motor and sensory neurons in these patients occurs either by damage to myelinating Schwann cells (CMT type 1, CMT1) or to axons (CMT type 2, CMT2). Differentiation of CMT subtypes can be achieved in electrophysiological examination of the patients where CMT1 patients show severe reduction in motor nerve conduction velocities (NCV < 38 m/s) while CMT2 patients display normal or slightly decreased NCV (>38 m/s) but a relatively low action potential amplitude (Azzedine et al., 2012; Bird, 2019). There are no significant differences in clinical features of the demyelinating and axonal forms of CMT. CMT symptoms ranges from muscle atrophy, areflexia, and food deformities to loss of pain and temperature sensation particularly in hands and feet (Szigeti & Lupski, 2009). The key feature of CMT symptoms is their symmetric manifestation.

Despite significant improvements in pathophysiological detection of CMT, however due to their late‐onset, gradual progression, and lack of clear family history as well as symptoms overlap with other neuropathies including motor neuron disease and chronic inflammatory demyelinating polyneuropathy (CIDP) correct diagnosis of CMT without genetic testing is often challenging (Auer‐Grumbach et al., 2016). In addition, variable expressivity in disease phenotypes including progression, severity and age of onset in different patients even among affected members of the same family with identical genetic defect further complicate the diagnosis process (Azzedine et al., 2012). However, a high genetic heterogeneity and a low genotype–phenotype correlation impede establishment of a specific genetic test for correct diagnosis of different subtypes of CMT. Until now more than 90 genes with different mode of inheritance have been associated to CMT (Bird, 2019). The genetic causes of more than 90% of CMT cases are due to mutations in four genes including *PMP22*, *GJB1*, *MFN2*, and *MPZ* (Auer‐Grumbach et al., 2003; Chung, 2006; Nave et al., 2007; Timmerman et al., 2014), with *PMP22* mutations alone accounting for ~70% of CMT1 cases while in CMT2, mutations in *GJB1* and *MFN2* are more frequent (Azzedine et al., 2012). The *MME* gene (OMIM #120520) is among frequently mutated genes in patients with late‐onset axonal form of CMT (Auer‐Grumbach et al., 2016; Higuchi et al., 2016; Hong et al., 2019; Lupo et al., 2018; Senderek et al., 2020; Taghizadeh et al., 2020). *MME* mutations have been shown to associate to both dominant and recessive CMT2. Dominantly inherited mutations show variable phenotypic features with no clear genotype–phenotype segregation pattern consistent with their incomplete penetrance (Auer‐Grumbach et al., 2016; Depondt et al., 2016).

The high clinical variability and low genotype–phenotype correlations suggest for the existence of other modifier environmental factors or genes. Genome wide association studies have identified several potential modifier loci for CMT1 including *LITAF* (Meggouh et al., 2005; Sinkiewicz‐Darol et al., 2015), *MIR149* (Nam et al., 2018), and *SIPA1L2* (Tao et al., 2019). The genetic basis for most late‐onset CMT type 2 cases is still unknown. Recent extensive use of whole genome and whole exome sequencing approaches for genetic testing of CMT patients expanded the number of genes identified to be implicated in CMT2.

In this report, we describe the clinical features and phenotypic variations of three members of a large consanguineous Iranian family diagnosed with CMT2. To identify the causative mutation, one of the patients was subjected to whole exome sequencing (WES) which resulted in the identification of a novel nonsense mutation (c.1564C>T; p.Q522X) in exon 16 of the *MME* gene (GenBank reference sequence: NM_000902) which was further confirmed in his parents, his affected brother, and the index case (his cousin).

## 2 MATERIAL AND METHODS

### 2.1 Study subjects

The cases included in this study are members of a large consanguineous family which show almost similar clinical presentations. Written informed consent for performing WES and publishing the report was obtained from the patients and their unaffected relatives during blood sampling.

### 2.2 Blood sampling and DNA extraction

Peripheral blood was collected in ethylenediaminetetraacetic acid tubes. DNA was extracted from 1 mL blood using RGDE method (Ali et al., 2008). The quality and quantity of the extracted DNA were evaluated using both spectrophotometry and agarose gel electrophoresis. DNA with concentrations >100 ng/μL and absorbance ratio 260/280 > 1.8 were sent for WES.

### 2.3 WES, variant calling and filtration

Library preparation was performed using Agilent SureSelect Human All Exon V6 (60 Mb) capture kit (Agilent). Exome sequencing was performed on an Ilumina HiSeq4000 sequencing platform (Illumina) generated ~14 Gbp of 150 bp paired‐end reads. Sequencing was carried out at the Beijing Genome Institute (BGI) sequencing center.

After screening for low quality sequences, the survived paired‐end reads were aligned to the human reference genome (GRCh38) using Burrows–Wheeler Aligner (BWA) in MEM mode (Li, 2013). The resulting SAM file was converted into BAM file format and sorted according to the sequence coordinates using Samtools v1.9. Marking PCR duplicates, base quality recalibration, indel realignment, and variant calling was performed using Genome Analysis Toolkit (GATK) v4.2.0.0. All variants with QD < 2.0, FS > 60.0, MQ < 40.0, SOR > 4.0, MQRankSum < −12.5, and ReadPosRankSum < −8.0 were filtered out. The remaining variants were functionally annotated using ANNOVAR (Wang et al., 2010) and further filtered based on their allele frequency, genomic position, functional impact on the protein, and the possible role of the gene in peripheral nervous system in VarAFT software (Desvignes et al., 2018). We first excluded intronic, intergenic, upstream, downstream, synonymous, and non‐frameshift variants and then filtered variants with minor allele frequency >1% based on genome aggregation database (genomAD, https://gnomad.broadinstitute.org/), exome aggregation consortium (ExomAC, https://gnomad.broadinstitute.org/), 1000 genome project (http://browser.1000genomes.org/), and Iranome databases (Fattahi et al., 2019). All the remaining variants were annotated for their functional consequences on the protein using UMD predictor, SIFT, PolyPhen2, Mutation Taster, Mutation Assessor, Provean, M‐CAP, and LRP. We also filtered variants with combined annotation‐dependent depletion (CADD) score (Rentzsch et al., 2019) < 15, DANN score (Quang et al., 2015) <0.9, Eigen score <1 and GERP++ score <2. The final list of candidate homozygous variants (43 variants in 37 genes) was manually filtered for nonrelevant splicing, ncRNA, 5′‐UTR, and 3′‐UTR variants resulting in five variants including four non‐synonymous exonic variants in MUC3A gene and a nonsense variant in *MME* gene (GenBank reference sequence: NM_000902).

### 2.4 Sanger sequencing

For confirmation of the candidate mutation in affected individuals, their unaffected relatives and non‐relatives, the genomic region containing mutation was PCR amplified and sequenced on an ABI 3500 Genetic analyzer (Applied Biosystems). The following MME_Fw 5′‐GGCTGGAATACTGACCTGTGATA‐3′ and MME_Rv 5′‐CTGATAGGCAATGACATAAGACGA‐3′ primers were used to amplify a 555‐bp fragment for Sanger sequencing.

## 3 RESULTS

**FIGURE 1:**
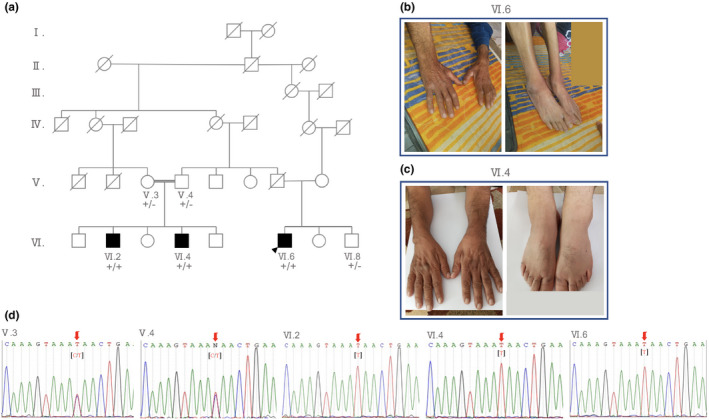
Family pedigree and photographs showing distal limbs in two affected members. (a) Pedigree and segregation analysis of *MME* (NCBI accession NM_000902) mutation in a consanguineous family with multiple affected patients diagnosed by late‐onset axonal Charcot–Marie–Tooth disease (CMT2). Affected members are homozygous for p.Q522* and unaffected members are either heterozygous or wild type. Circles represent women and squares represent men. Filled squares indicate affected individuals. Black arrow indicates the proband. +/+ indicates homozygous for mutation and +/− indicates heterozygous for mutation. (b) Muscle atrophy and deformities in hands and feet in the proband (patients VI.6). (c) Photographs show distal muscle wasting in hands and feet in patient VI.4. (d) Sanger sequencing electrophoregrams show the genotype of two affected siblings (VI.2 and VI.4), their unaffected parents (V.3 and V.4), and their affected cousin (VI.6)

Here, we studied a family with multiple affected cases diagnosed with late‐onset CMT2 each showing different levels of disease severity (Figure 1a). The index case (VI.6) was at the age 45 when he complained for the first time for weakness in feet and hands and difficulty in walking. The disease symptoms were initially detected in his feet and subsequently in his hands. Initial examination by a neurologist revealed higher involvement of distal and lower limbs and motor neurons compared to sensory neurons (Table 1). Electromyography (EMG) study revealed spontaneous activity and a reduced recruitment pattern. It also showed grade 2+ positive sharp waves in the tibialis anterior and flexor carpi radialis muscles bilaterally and grade 1+ in the gastrocnemius, first dorsal interosseous, and vastus medialis muscles. Motor nerve conduction velocity (MCV) analysis showed a reduced amplitude in both median and tibial nerves and a relatively normal velocity (Table 2). An increased latency was noted in median and ulnar nerve on both sides. The absence of compound muscle action potential (CMAP) was also noticeable in both tibial and peroneal nerves. Testing for sensory nerve conduction velocity (SCV) revealed the absence of sensory nerve action potential (SNAP) in both upper and lower extremities. The electrophysiological and nerve conduction findings were consistent with chronic axonal sensorimotor polyneuropathy (predominantly motor) with active denervation. A possible diagnosis of motor neuron disease or a type of chronic inflammatory demyelinating polyneuropathy (CIDP) was considered. However, testing for high protein level in cerebrospinal fluid (CSF) which associates with CIDP with almost similar clinical presentation to CMT was negative. Magnetic resonance imaging (MRI) of brain and spine was also unremarkable.

**TABLE 1:** Patient clinical features and their neurological findings

Characteristics	Patient VI.2	Patient VI.4	Patient VI.6
Gender	Male	Male	Male
Age (years)	52	51	52
Age at onset (years)	48	46	45
Clinical features	Muscle weakness, gait difficulty, paresthesia in fingers	Muscle weakness and atrophy in distal part of hands and feet, gait difficulty, frequent fall	Muscle weakness and atrophy in upper and lower limbs, gait difficulty, frequent fall, impaired temperature and pain sensation in extremities
Foot deformity	Foot drop	Bilateral foot drop	Bilateral food drop and hammer toe
Additional clinical features	—	stuttering	—
NCV/EMG findings	Lower > upper Distal > proximal Motor > sensory	Lower > upper Distal > proximal Motor > sensory	Lower > upper Distal > proximal Motor > sensory
Diagnosis suggested based on NCV/EMG	Chronic demyelinating sensorimotor polyneuropathy, CMT	Axonal demyelinating sensorimotor polyneuropathy, CIPD	Paraneoplastic motor neuron disease, CIPD
Brain and spinal MRI imaging	Normal MRI	Multiple high signal foci in centrum semiovale and subcortical area of cerebral hemisphere	Normal MRI
Genetic test performed before WES	—	—	PMP22 duplication/deletion
Clinical diagnosis	CMT	CMT	CMT

Abbreviations: CIPD, chronic inflammatory demyelinating polyneuropathy; CMT, Charcot–Marie–Tooth disease; EMG, electromyography; MRI, magnetic resonance imaging; NCV, nerve conduction velocity; WES, whole exome sequencing.

**TABLE 2:** Electrophysiological and nerve conduction study of motor and sensory neurons in patients with type 2 Charcot–Marie–Tooth disease (CMT2)

Patient	Nerve	Stimulation site	Side	Recoding site	Latency (ms)	Amplitude (μV)	Conduction velocity (m/s)
*MCV*							
Patient VI.2	Median	Wrist	R	APB	3.80	4.83	
			L		4.20	4.13	
		Elbow	R	APB	9.10	4.81	35.85
			L		9.70	3.29	38.18
	Ulnar	Wrist	R	ADM	3.30	5.26	
			L		2.60	4.25	
		Elbow	R	ADM	7.20	4.94	42.59
			L		6.80	3.55	42.86
	Tibial	—	R	Knee	NR	0.00	
			L		7.50	0.00	
Patient VI.4	Median	Wrist	R	APB	4.38	0.91	
			L		5.58	0.39	
		Elbow	R	APB	12.83	0.31	NR
			L		14.05	0.39	NR
	Ulnar	Wrist	R	ADM	3.93	0.74	
			L		3.08	2.19	
		Elbow	R	ADM	9	0.72	NR
			L		9.28	1.39	NR
	Tibial	—	R	Knee	15.35	0.19	
			L		0.7	0.31	
Patient VI.6	Median	Wrist	R	APB	3.44	6.5	
			L		3.23	5.2	
		Elbow	R	APB	9.06	4.8	48
			L		9.48	3.8	43
	Ulnar	Wrist	R	ADM	2.60	4.3	
			L		2.66	3.1	
		Elbow	R	ADM	8.18	4.2	48
			L		7.81	3.1	50
	Tibial	—	R	—	NR	NR	
			L	—	NR	NR	
*SCV*							
Patient VI.2	Median	Wrist	R	Digit II	3.90	14.11	NR
			L		3.90	12.25	NR
	Ulnar	Wrist	R	Digit V	3.20	20.71	NR
			L		3.5	9.54	NR
	Sural	Ankle	R	—	16.40	0.00	NR
			L	—	10.0	0.00	NR
Patient VI.4	Median	Wrist	R	Digit III	3.7	6.21	32.43
			L		4.33	4.39	27.75
	Ulnar	Wrist	R	Digit V	3.5	9.25	34.29
			L		NR	NR	NR
	Sural	—	R	—	NR	NR	NR
			L	—	NR	NR	NR
Patient VI.6	Median	Wrist	R	Digit II	3.13	6.7	42
			L		2.92	9.6	45
	Ulnar	Wrist	R	Digit V	NR	NR	NR
			L		2.71	10.8	41
	Sural	Ankle	R		3.65	3.5	NR
			L		3.02	3.4	58

Abbreviations: ADM, abductor digiti minimi; APB, abductor pollicis brevis; L, left; MCV, motor nerve conduction velocity; NR, not recorded; R, right; SCV, sensory nerve conduction velocity.

With no effective cure, the disease progressed gradually and the patient further suffered from extensive muscular atrophy in both lower and upper limbs (Figure 1b). He currently shows typical symptoms of CMT including walking difficulty, bilateral foot drop, steppage gait, foot deformities (hammer toe), and loss of pain and temperature sensation in the lower parts of hands and feet. The patient's father died 15 years ago at the age of 58 from cancer. He did not show any similar phenotype. His mother is alive (73 years old), cognitively healthy and do not show any similar clinical features. With the development of the same clinical symptoms in his first cousins we offered WES for one of the affected members of the family (patient VI.4) who had most similar clinical presentations to the proband.

Patient VI.4 is a 51 years old man born to healthy consanguineous parents (Figure 1a). He suffers from weakness in hands and feet, difficulty in walking and noticeable muscular atrophy particularly in hands (Table 1). Early sign of weakness and walking difficulty was noted at the age of 45. His major problem is foot drop, frequent fall, gait steppage, and impaired sensation of temperature and pain in his fingers. Figure 1C shows the extent of muscle atrophy in patient hands and feet 4 years after disease manifestation. He also suffers from severe stuttering with frequent repetition of sounds and difficulty in fluent speech. Neurophysiological examination revealed a decreased CMAP in median nerve on both sides and an increased latency in the same nerve in both motor and sensory examinations (Table 2). The ulnar nerve displayed a normal CMAP and SNAP. The bilateral absence of CMAP and SNAP in both tibial and peroneal nerves was also noticeable. Based on these findings, a neurologist concluded a form of axonal‐demyelinating sensorimotor polyneuropathy. He also considered for CIDP; however, CSF analysis was normal. Brain MRI imaging showed the existence of multiple high signal foci in centrum semiovale and subcortical area of cerebral hemisphere consistent with demyelination or chronic ischemic changes. Spinal cord imaging showed discovertebral degenerative changes in cervical regions consistent with disk dehydration. Disk space narrowing and marginal spur formation were also noted. His parents do not show any symptoms of the disease supporting a recessive mode of inheritance. His mother currently suffers from moderate Alzheimer disease. His maternal uncle (died at 77) also suffered from polyneuropathy and was unable to walk independently after the age 65. Based on clinical presentations and family history we finally decided to perform WES for this patient.

Patient VI.2 is a 52 years old man who showed early symptoms of the disease at the age of 48. He suffers from gait and walking difficulty and displays muscle weakness and moderate muscle atrophy in both upper and lower limbs. He also complains of paresthesia in his fingers. Brain and spinal cord MRI imaging was normal. Electromyography study revealed spontaneous activity in first dorsal interosseous, tibial anterior, vastus medialis muscles on both sides (Table 1). A reduced recruitment pattern was also recorded for these muscles. For most tested muscles an increased duration (grades 2+ and 3+) and amplitude (grade 3+) along with polyphasia was recorded under motor unit action potential (MUAP) indicating moderate to severe peripheral neurogenic impairment. NCV analysis showed a bilateral increase in latency and decrease in velocity in median and ulnar nerves (Table 2). Based on these findings a neurologist concluded for chronic demyelinating sensorimotor polyneuropathy with evidence of axonal loss. A type CIDP or CMT was considered. CIDP diagnosis was rejected upon lumbar puncture test. However, based on family history and the presentation of the similar clinical features in his brother, CMT was considered more likely.

**FIGURE 2:**
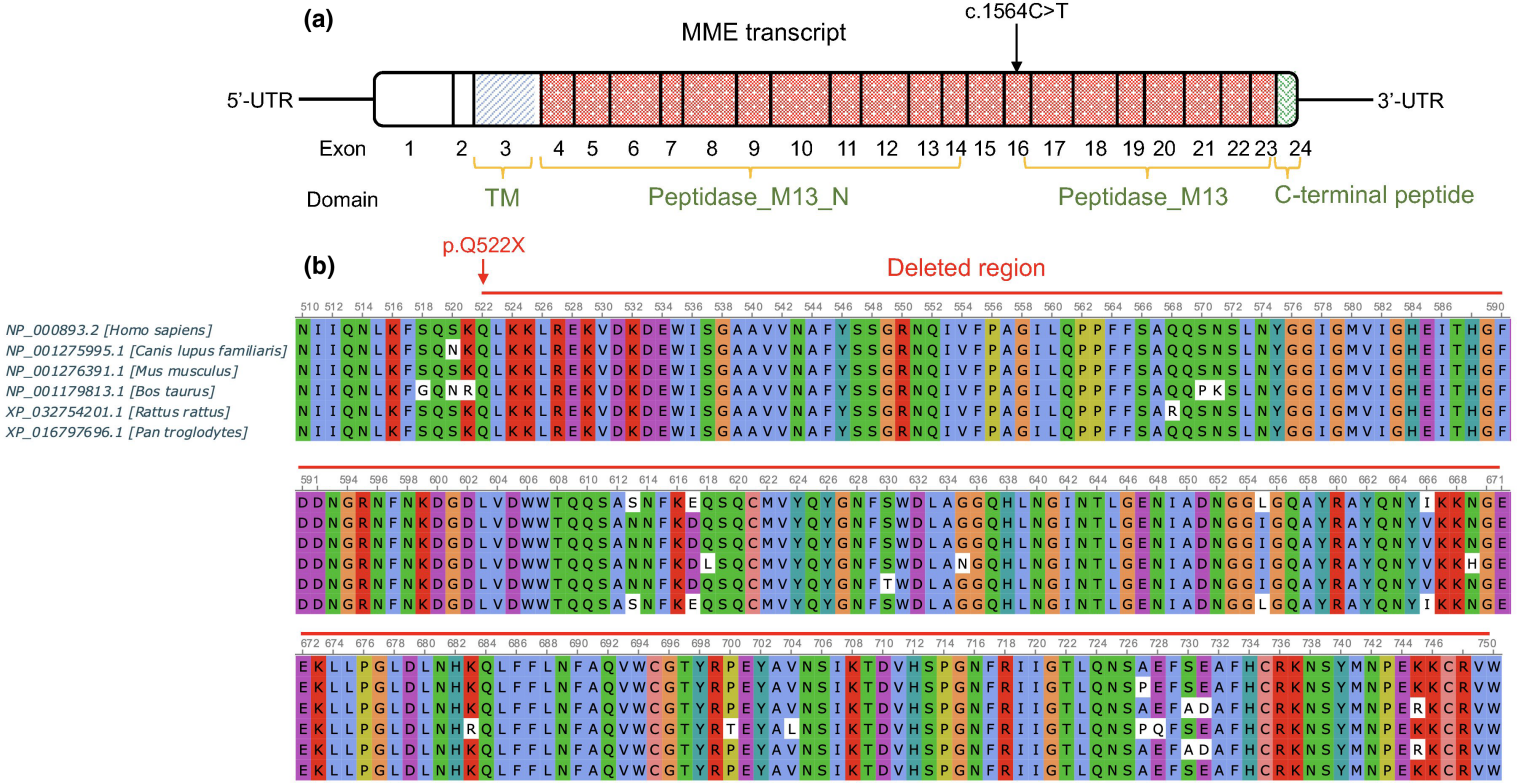
(a) Schematic representation of exons in the *MME* transcript (NM_000902) and the localization of domains in the encoded neprilysin protein (UniProt accession number P08473). The position of the identified mutation (c.1564C>T) was shown by a vertical arrow. (b) The effect of stopgain mutation p.Q522* depicted in multiple sequence alignment of the human neprilysin protein with its homologs in dog (*Canis Lupus familiaris*), mouse (*Mus musculus*), cow (*Bus taurus*), rat (*Rattus rattus*), and chimpanzee (*Pan troglodytes*). The p.Q522* mutation causes a complete loss of c‐terminus end of the neprilysin protein which contains zinc‐binding and the enzyme active sites

For all patients included in this study routine laboratory tests including CBC, urine analysis, serum biochemistry, autoimmune disorder screening was performed but no significant findings were reported. None of the patients have any cognitive impairment. Based on the family pedigree and similar neurophysiological findings characteristic of CMT disease, an autosomal recessive CMT was finally considered. None of the patients are still wheelchair dependent and thus their CMT phenotype is scored moderate. Due to the heterogenic nature of the disease we decided to whole exome sequence one of the affected members of the family (patient VI.4) whose parents are alive. Before WES, we found that the proband had previously been tested for *PMP22* duplication/deletion but no significant finding was reported. WES resulted in identification of a novel nonsense mutation in membrane metalloendopeptidase (*MME*, NM_000902) gene which encodes for a type II transmembrane glycoprotein neprilysin (NEP). The underlying mutation was a substitution of C by T (chr3:155148616, Genome version GRCh38) in exon 16 (c.1564C>T) of the *MME* gene which converted a glutamine codon to a termination codon (p.Q522*). The mutation causes a complete loss of peptidase_M13 domain from the C‐terminal region of NEP protein (Figure 2a). The deleted region is highly conserved among mammalians and thought to play a significant role in NEP function (Figure 2b). The candidate variant has a CADD score 45, DANN score 0.998, and GERP++ 5.93 and predicted to be deleterious based on Mutation Taster and LRT. The variant has not been reported in dbSNP build 150 (http://www.ncbi.nlm.nih.gov/SNP/), ExAC, 1000 genomes project, and gnomAD databases. The variant was also absent in 800 healthy individuals from eight Iranian ethnicity groups sequenced in the Iranome project (Fattahi et al., 2019). Sanger sequencing confirmed the co‐segregation of the mutation with the disease phenotype in the family (Figure 1D). All affected members of the family were found homozygous for this mutation (Figure 1D). The parents (V.3 and V.4) of the affected siblings (VI.2 and VI.4) and the unaffected proband's brother (VI.8, 39 years old) were heterozygote for the mutation, concordant with the autosomal recessive mode of inheritance. The variant was submitted to the ClinVar database as a pathogenic variant under the accession number SCV001469087.1.

## 4 DISCUSSION

Here, we studied a large consanguineous six generations family with multiple affected patients suffering from a range of phenotypes including muscle weakness, walking difficulty, foot drop, muscular atrophy, and impaired temperature and pain sensation particularly in upper and lower extremities. Symptoms were initially started in the lower limbs but subsequently extended to the upper extremities. In electrophysiological examinations, patients manifested a relatively unique phenotypic features with evidence of axonal damage to motor and sensory nerves characteristics of a late‐onset axonal form of polyneuropathy. In NCS, a normal nerve conduction velocity (>38 m/s) and a low action potential in ulnar and median nerves were consistent with an axonal form of CMT (CMT2). Due to an extensive symptom overlap with CIDP, all patients were subjected to CSF analysis but no remarkable finding was reported. Based on the clinical findings and family pedigree, a muscular neurologist finally suggested genetic testing for CMT disease. To identify the underlying mutation, we therefore carried out WES in one of the patients who born to healthy consanguineous parents. All patients were in the same age range (±2 years) when they showed early symptoms of the disease. Due to the late age of onset (>45 years) other affected persons may also present in this family.

Exome sequencing identified a homozygous mutation, c.1564C>T (p.Gln522*), in *MME* gene as the genetic cause for this disease condition. Sanger sequencing of the mutation confirmed co‐segregation with the disease in the family. *MME* gene (3q25.2) has 24 exons which encodes for a highly conserved zinc‐dependent membrane metallopetidase (neprilysin, NEP) which structurally consists of a transmembrane domain (TM, exon 3), a peptidase_M13 domain (exons 4–23) and a 32‐aa carboxyterminal peptide (exon 24) (Bayes‐Genis et al., 2016; D'Adamio et al., 1989). Since zinc binding and catalytic sites are located in exon 19 (D'Adamio et al., 1989), the c.1564C>T (p.Gln522*) mutation (in exon 16) causes a complete loss of C‐terminal region of the peptidase_M13 catalytic domain. The candidate mutation is thus a loss‐of‐function (LoF) mutation which causes either protein truncation or mRNA degradation by the nonsense‐mediated mRNA decay. NEP is a cell surface ectoenzyme catalyzing the degradation of diverse peptide substrates outside of cells (Nalivaeva et al., 2020). NEP inactivates a wide range of peptide substrates including enkephalins, neurokinin A, substance P, bradykinin, endothelins, somatostatin, adrenomedullin, bombesin‐like peptides, glucagon, thymopentin as well as the amyloid‐β (Aβ) which accumulates in Alzheimer's disease (AD) (Nalivaeva et al., 2020).

In previous study, *MME* was among frequently mutated genes in Iranian patients with hereditary polyneuropathies (Taghizadeh et al., 2020). Interestingly, two homozygous mutations c.1342C>T (p.Arg448*) and c.1861T>C (p.Cys621Arg) detected in *MME* were associated to CMT2 in two unrelated Iranian families, both of which were predicted to be pathogenic based on in silico analysis. Interestingly, p.Arg448* was also detected in homozygous status in Spanish patients diagnosed with late‐onset axonal CMT (Lupo et al., 2018) and in compound heterozygote in Chinese patients with distal hereditary motor neuropathy (Hong et al., 2019). While in the study by Auer‐Grumbach et al. (2016) this mutation was associated with late‐onset autosomal dominant polyneuropathies.

Previous gene burden analysis revealed a significant association of *MME* LoF mutations with late‐onset peripheral neuropathies (Auer‐Grumbach et al., 2016). Particularly, *MME* LoF mutations associate with a reduced NEP abundance in adipose tissue and blood samples obtained from CMT patients, suggesting a haploinsufficiency mechanism consistent with an autosomal dominant mode of inheritance (Auer‐Grumbach et al., 2016). However, we showed that obligate heterozygote carries are healthy and do not show any sign of disease consistent with findings of Higuchi et al. (2016) in Japanese families with *MME* mutations further supporting that haploinsufficiency is an unlikely mechanism for the development of CMT2. Dominantly inherited mutations in *MME* gene (p.C143Y) have also been associated to neuropathies with cerebral involvement (spinocerebellar ataxia) (Depondt et al., 2016). Recently, Lupo et al. (2018) reported that homozygous and compound heterozygous mutations in *MME* gene associate with the late‐onset axonal neuropathy (CMT2). Patients carrying these mutations showed similar clinical and phenotypic features in line with our findings. However, they noted that patients with heterozygote mutations display phenotypic variations with no apparent co‐segregation pattern with the disease and thus may not cause the neuropathy phenotype. These findings suggested that heterozygote mutations in *MME* gene can associate with peripheral neuropathies with varying clinical presentations but likely in the presence of other modifying factors or genes.

The exact mechanisms of NEP mutations in the development of CMT disease have remained elusive. Studies in mice knocked out for *MME* showed impaired myelination of Remak bundles indicating a regenerative role for NEP in peripheral nerves (Auer‐Grumbach et al., 2016). In peripheral nervus, NEP is largely localized to the myelinating Schwan cells (Kioussi & Matsas, 1991). The expression of NEB has been reported in myelin sheath in sural nerves biopsies obtained from healthy individuals but its expression was absent or significantly reduced in CMT patients with *MME* LoF mutations (Higuchi et al., 2016). Since CMT type 2 is caused by axonal injuries, NEP inactivating mutations likely disrupt myelinating function of Schwan cells and causing axonal injuries to peripheral nerves. The upregulation of NEP following axonal injury to nerves in rats suggested that it is involved in neuronal regeneration by degrading inhibitory peptides (Kioussi et al., 1995).

In addition to its key role in neuronal development and regeneration, NEP has been shown to play an important role in degradation of Aβ peptides (Park et al., 2013). An overexpression of NEP in neurons associated with decreased Aβ level, reduced Aβ plaque formation and increased survival rate in transgenic mouse model of AD (Leissring et al., 2003). It has been shown that NEP‐mediated processing of neuropeptide Y to C‐terminal fragment also contributes to the protection of neurons from neurodegenerating effect of Aβ (Rose et al., 2009). In human, mutations in the *MME* gene have not been associated to familial form of AD. However, there are several conflicting reports that show the association with sporadic form of AD (Chen et al., 2020; Wood et al., 2007; Zhang et al., 2017). Since c.1564C>T mutation in *MME* is a LoF mutation, it could be speculated that CMT patients are susceptible to AD. However, none of the patients had cognitive impairment except for obligatory carrier V.3 (76 years old women) who currently suffers from AD. Since AD is commonly developed in elderly people (age of onset >60), failure to detect disease phenotype in these patients might be due to their low age. In line with our findings, Higuchi et al. (2016) also reported that patients with LoF mutations in *MME* gene did not show evidence for Aβ accumulation and AD, suggesting that the absence of NEP in these patients is compensated by other Aβ degrading enzyme systems. The development of AD in CMT patients carrying *MME* LoF mutations has been reported at least in one individual with homozygous p.Pro156Leufs*14 mutation in the study cohort from Spain (Lupo et al., 2018), although the AD phenotype manifested three decades after the CMT2 diagnosis.

In conclusion, we identified a LoF mutation in *MME* gene (p.Q522*) which caused late‐onset axonal form of CMT. The mutation was shown to co‐segregate with the disease in the family in an autosomal recessive mode of inheritance. All affected members of the family showed similar clinical and phenotypic features with no evidence of cognitive dysfunction supporting the finding that *MME* mutations are not sufficient for the development of early‐onset AD. Further research would be required to unravel the mechanistic function of *MME* mutations in the development of late‐onset sensorimotor polyneuropathies. Establishment of genetic tests for screening mutations in *MME* gene could be considered as a primary option for the diagnosis of late‐onset axonal form of CMT or CMT2.

## CONFLICT OF INTEREST

The authors have no conflict of interest to disclose.

## AUTHOR CONTRIBUTION

ZJ and RK performed the research, EK extracted genomic DNA for exome sequencing, MMH contributed in study design and conception, JG contributed in study design and conception, analysis and interpretation of the exome sequencing data, and wrote the manuscript. All authors read and approved the final manuscript.

## ETHICAL COMPLIANCE

The study was conducted in accordance to the ethical guidelines of the World Medical Association Declaration of Helsinki and approved by the ethic committee of Baqiyatallah University of Medical Sciences.

## Data Availability

The raw sequence data that support the findings of this study are openly available in NCBI short read archive under BioProject ID PRJNA747680 and accession number SRR15179658.
